# Age-Specific ADME Gene Expression in Infant Intestinal Enteroids

**DOI:** 10.1021/acs.molpharmaceut.4c00302

**Published:** 2024-08-09

**Authors:** Eva J. Streekstra, Tom Scheer-Weijers, Michael Bscheider, Sabine Fuerst-Recktenwald, Adrian Roth, Sven C. D. van Ijzendoorn, Sanne Botden, Willem de Boode, Martijn W. J. Stommel, Rick Greupink, Frans G. M. Russel, Evita van de Steeg, Saskia N. de Wildt

**Affiliations:** † Department of Pharmacy, Division of Pharmacology and Toxicology, Radboud University Medical Center, Nijmegen 6525GA, The Netherlands; ‡ Department of Metabolic Health Research, Netherlands Organization for Applied Scientific Research (TNO), Leiden 2333BE, The Netherlands; § F. Hoffmann-La Roche Ltd, Basel CH-4070, Switzerland; ∥ Department of Biomedical Sciences, University of Groningen, University Medical Center Groningen, Groningen 9713GZ, The Netherlands; ⊥ Department of Pediatric Surgery, Radboud University Medical Center, Amalia Children’s Hospital, Nijmegen 6525GA, The Netherlands; # Department of Pediatrics, Division of Neonatology, Radboud University Medical Center, Amalia Children’s Hospital, Nijmegen 6525GA, The Netherlands; ¶ Department of Surgery, Radboud University Medical Center, Nijmegen 6525GA, The Netherlands; ∇ Department of Intensive Care, Radboud University Medical Center, Nijmegen 6525GA, The Netherlands; ○ Department of Neonatal and Pediatric Intensive Care, Erasmus MC Sophia Children’s Hospital, Rotterdam 3015GD, The Netherlands

**Keywords:** intestine, intestinal organoids, enteroids pediatrics, pharmacokinetics, drug transporters, drug metabolizing enzymes

## Abstract

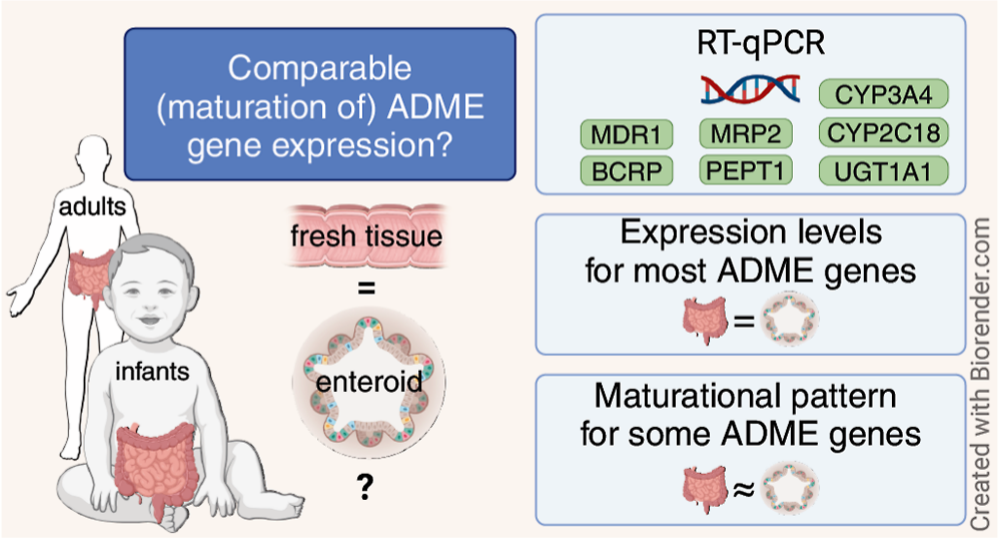

In childhood, developmental changes and environmental interactions highly affect orally dosed drug disposition across the age range. To optimize dosing regimens and ensure safe use of drugs in pediatric patients, understanding this age-dependent biology is necessary. In this proof-of-concept study, we aimed to culture age-specific enteroids from infant tissue which represent its original donor material, specifically for drug transport and metabolism. Enteroid lines from fresh infant tissues (*n* = 8, age range: 0.3–45 postnatal weeks) and adult tissues (*n* = 3) were established and expanded to 3D self-organizing enteroids. The gene expression of drug transporters P-gp (*ABCB1*), BCRP (*ABCG2*), MRP2 (*ABCC2*), and PEPT1 (*SLC15A1*) and drug metabolizing enzymes CYP3A4, CYP2C18, and UGT1A1 was determined with RT-qPCR in fresh tissue and its derivative differentiated enteroids. Expression levels of P-gp, BCRP, MRP2, and CYP3A4 were similar between tissues and enteroids. PEPT1 and CYP2C18 expression was lower in enteroids compared to that in the tissue. The expression of UGT1A1 in the tissue was lower than that in enteroids. The gene expression did not change with the enteroid passage number for all genes studied. Similar maturational patterns in tissues and enteroids were visually observed for P-gp, PEPT1, MRP2, CYP3A4, CYP2C18, and VIL1. In this explorative study, interpatient variability was high, likely due to the diverse patient characteristics of the sampled population (e.g., disease, age, and treatment). To summarize, maturational patterns of clinically relevant ADME genes in tissue were maintained in enteroids. These findings are an important step toward the potential use of pediatric enteroids in pediatric drug development, which in the future may lead to improved pediatric safety predictions during drug development. We reason that such an approach can contribute to a potential age-specific platform to study and predict drug exposure and intestinal safety in pediatrics.

## Introduction

The intestinal tract is a specialized organ for the disposition of nutrition and orally dosed drugs. In childhood, developmental changes and environmental interactions drive the maturation of the involved processes, e.g., expression of transport protein or metabolizing enzymes.^1^ To optimize dosing regimens and ensure safe usage of drugs in pediatric patients, understanding this age-dependent biology is necessary.

Key hepatic metabolic enzymes including cytochrome P450s (CYPs) and uridine diphosphate glucuronosyltransferases (UGTs) have distinct expression and activity profiles in children as compared to adults.^2,3^ Liver in vitro data are successfully applied in physiologically based pharmacokinetic (PBPK) models to predict pediatric pharmacokinetics. Contrastingly, data on ontogeny driving intestinal metabolic function are sparser, with RNA and protein expression data indicating age-related expression differences as well.^4,5^ However, functional pediatric data are largely lacking, as appropriate experimental intestinal models are challenging to conduct. As an example, while Ussing chambers are useful to study active drug transport and metabolism,^6^ fresh tissue samples from young patients are often too small for this method. Furthermore, drug disposition occurs in specific gut segments, while other segments fulfill distinct functions.

Here, stem-cell-derived enteroids hold a big promise as they allow functional assays of the epithelium; they are derived from postnatal, tissue-resident stem cells and can be expanded from the tiniest biopsies.^7,8^ Enteroids retain genomic information and the derived functional capacity, e.g., in cystic fibrosis therapy response.^9^ Furthermore, unlike induced pluripotent stem cell (iPSC)-derived intestinal organoids,^10−12^ postnatal enteroids have the potential to retain age- and gut segment-related biological characteristics, orchestrated by epigenetic regulation like methylation patterns that shift during childhood.^13,14^ Previous work showed that age-specific methylation patterns of intestinal epithelium are conserved in enteroids from children between 4 and 15 years, while prebirth, fetal enteroids (8–12 weeks of gestational age) do not display such age-induced methylation patterns.^15^ Therefore, enteroids may allow for a valuable preclinical human pediatric model for drug metabolism. As a major shift in ontogeny- and exposure-induced epigenetic regulation occurs soon after birth,^16^ we hypothesize that enteroids derived from infants, specifically at this early age range, and from intestinal gut segments with drug uptake will mirror the pharmacokinetic gene, protein, and activity patterns that are induced by early life epigenetic maturation.

In this proof-of-concept study, we aim to culture segment-specific enteroids (i.e., ileum) from infant tissue which retain age-specific properties representative to the original donor material, specifically for drug transport and metabolism. We reason that such an approach can contribute to a potential age-specific in silico platform to study and predict drug exposure and intestinal safety in pediatrics. Thus, we explored whether enteroids could be applied as an additional tool during drug development.

## Methodology

### Human Tissue

Pediatric residual ileum tissue was collected during elective and emergency surgery conducted at the Radboud University Medical Center (Radboudumc) in Nijmegen, The Netherlands, between August 2022 and August 2023. We obtained informed consent from parents and legal guardians for the use of residual material and access to clinical data. A waiver for formal ethical approval was given by the ethical committee of Radboudumc in accordance with the Dutch Law on Human Research. Adult terminal ileum tissues were derived from colorectal cancer patients who underwent (hemi)colectomy surgeries.^17^ No explicit informed consent was required for the utilization of anonymous residual material for research purposes in compliance with the Dutch Code of Conduct for Responsible Use.

### Tissue Handling

After surgical resection, the fresh tissue was directly put in an ice cold Krebs buffer and transported to the laboratory (within 15 min). For the neonates and infants, the sizes of the tissue pieces ranged from the size of a grain of rice to 1 cm^2^. If possible, the mucosa was separated from the submucosal layers and a part of the tissue was snap frozen in liquid nitrogen for RNA isolation within 1 h after surgical resection. For enteroid isolation, the rest of the tissue was stored overnight in a storage buffer (Table S1) on ice, and within 24 h, intestinal crypts were isolated to form enteroids. Alternatively, mucosal fragments were frozen at −80 °C in a Recovery Cell Culture Freezing medium (Gibco) for a period of <1 week for later enteroid isolation. When the tissue was collected outside office hours (samples 1, 3, and 4), it was directly put in a cold Krebs buffer (4 °C) after resection and stored overnight at 4 °C.

### 3D Human Intestinal Enteroids

3D human enteroids were cultured according to procedures provided by STEMCELL Technologies based on established protocols available in the literature.^7,18^ In brief, the mucosa layer was cut in 2–3 mm^2^ tissue segments which were thoroughly washed in a wash buffer (details on the composition of media and buffers used can be found in Table S1). Tissue fragments were subjected to a 1 h incubation in a crypt release solution at room temperature. Crypt fractions were collected in a crypt collection buffer. The acquired crypts were diluted in a 70–90% Matrigel solution and plated in 30 μL domes in 48-well plates. The domes were covered with 300 μL of IntestiCult human Organoid Growth Medium (OGM) and placed at 37 °C in a humidified incubator in the presence of 5% CO_2_. The culture medium was refreshed every 2–3 days. Enteroids were passaged every 7–10 days if not used for differentiation. After 3–5 days, the OGM was replaced by IntestiCult human Organoid Differentiation Medium (ODM). Differentiation took 3–4 days after which three organoid domes per vial were lysed and stored (−80 °C) until RNA isolation. Before every media change or passage, enteroids were evaluated visually via phase contrast microscopy.

### Reverse Transcription-Quantitative Polymerase Chain Reaction

Stored enteroid and tissue samples were thawed, and RNA was isolated with the Qiagen RNeasy mini kit according to the protocol. All RNA isolates were tested for integrity by gel electrophoresis. Afterward, we used an in-house protocol containing 5x strand buffer, dNTPs, random primer, DTT, RNAsin, and M-MLV reverse transcriptase for cDNA synthesis or RT-PCR super mix (Bio-Rad). qPCR was performed using TaqMan Universal PCR Master Mix, primers, probes, and its protocol on a CFX Connect Real-Time System (Bio-Rad). We investigated the relative gene expression of seven ADME-related genes in enteroid cells and their respective tissues. Selected genes play a major role in drug metabolism or transport: *ABCB1* (P-gp), *ABCG2* (BCRP), *ABCC2* (MRP2), *SLC15A1* (PEPT1), CYP3A4, UGT1A1, and CYP2C18. The primer probes used can be found in Table 1. The data were analyzed using the delta Ct method and expressed as a relative expression with 2^–ΔCt^ or fold change with the ΔΔCt method. We used Villin-1 to normalize the target genes for the enterocyte input of tissue and enteroids,^4,5,19−21^ as tissue biopsies used for RNA isolation still consist of a lamina propria in contrast to 3D enteroids, which only consist of the epithelial cell layer. Comparisons for Villin-1 were corrected for reference gene 18S to show stability with age and passage number.

**Table 1:** TaqMan Prime Probe Sets Used in This Study for Each Gene

gene	TaqMan primer probe
Villin-1	Hs01031739_m1
*ABCB1* (P-gp)	Hs01067802_m1
*ABCG2* (BCRP)	Hs00184979_m1
CYP3A4	Hs00604506_m1
UGT1A1	Hs02511055_s1
*SLC15A1* (PEPT1)	Hs00192639_m1
CYP2C18	Hs00426403_m1
*ABCC2* (MRP2)	Hs00166123_m1
18S	Hs99999901_m1

### Statistical Analysis

Data were summarized using descriptive statistics presented in the median ± inter quartile range. To evaluate the stability of the gene expression during enteroid culture, enteroids were divided into passage timeframes, i.e., earlier passage and late passage, with at least two passage differences. If more than one passage number was in the earlier or late group, the median relative expression was determined. Expression levels between passage numbers were compared with a Wilcoxon matched-pair signed rank test (Figure 2). When the expression did not differ significantly between passages, median gene expression was determined of all available passages per enteroid line. To compare median relative expression in paired tissue and enteroids, a Wilcoxon matched-pairs signed rank test was performed (Figure 3). Statistical analyses were performed using GraphPad Prism 8. *P*-values <0.05 were considered statistically significant.

**Figure 1:**
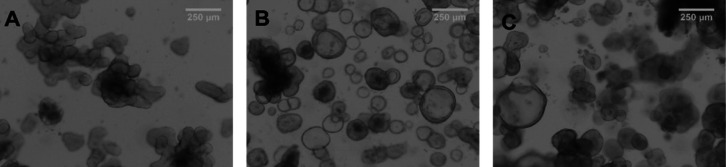
Representative phase-contrast microscopy pictures of 3D enteroids at passage 3 from a donor of 4.5 weeks old (A), at passage 8 from a 13 week-old donor (B), and an adult donor at passage 6 (C).

**Figure 2:**
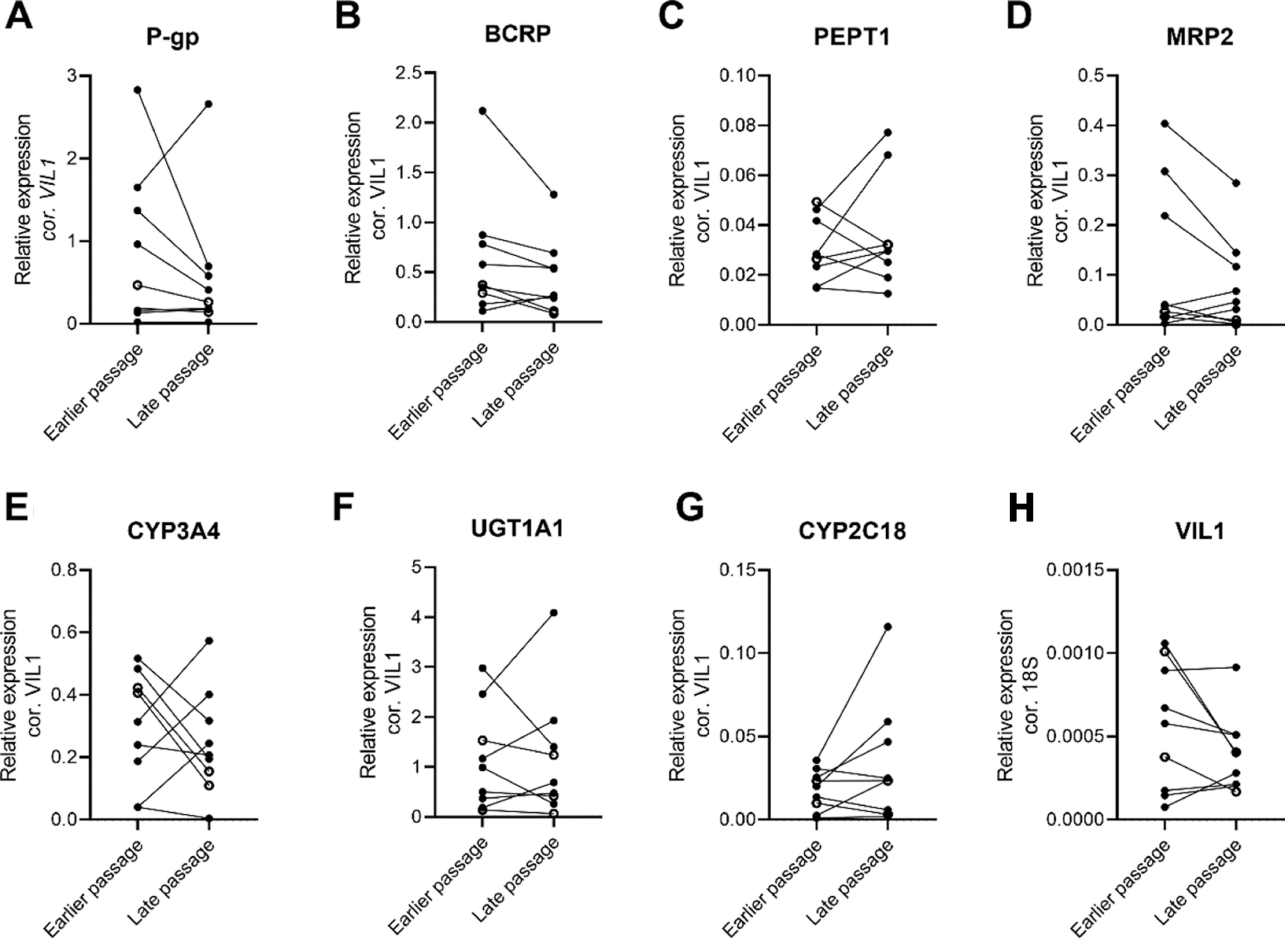
Gene expression in pediatric and adult enteroids at earlier and late passage numbers (with at least 2 passage number difference) corrected for Villin-1 shows no effect of passage number. Villin-1 corrected for 18S. Individual dots represent individual enteroid donors, open dots indicate adult donors, and closed dots represent pediatric donors. (A) P-gp (*ABCB1*), (B) BCRP (*ABCG2*), (C) PEPT1 (*SLC15A1*), (D) MRP2 (*ABCC2*), (E) CYP3A4, (F) UGT1A1, (G) CYP2C18, and (H) Villin-1. Donors included in this graph and corresponding passage numbers are presented in Table 2. Exact *p*-values and median values per group are presented in Table S2.

**Figure 3:**
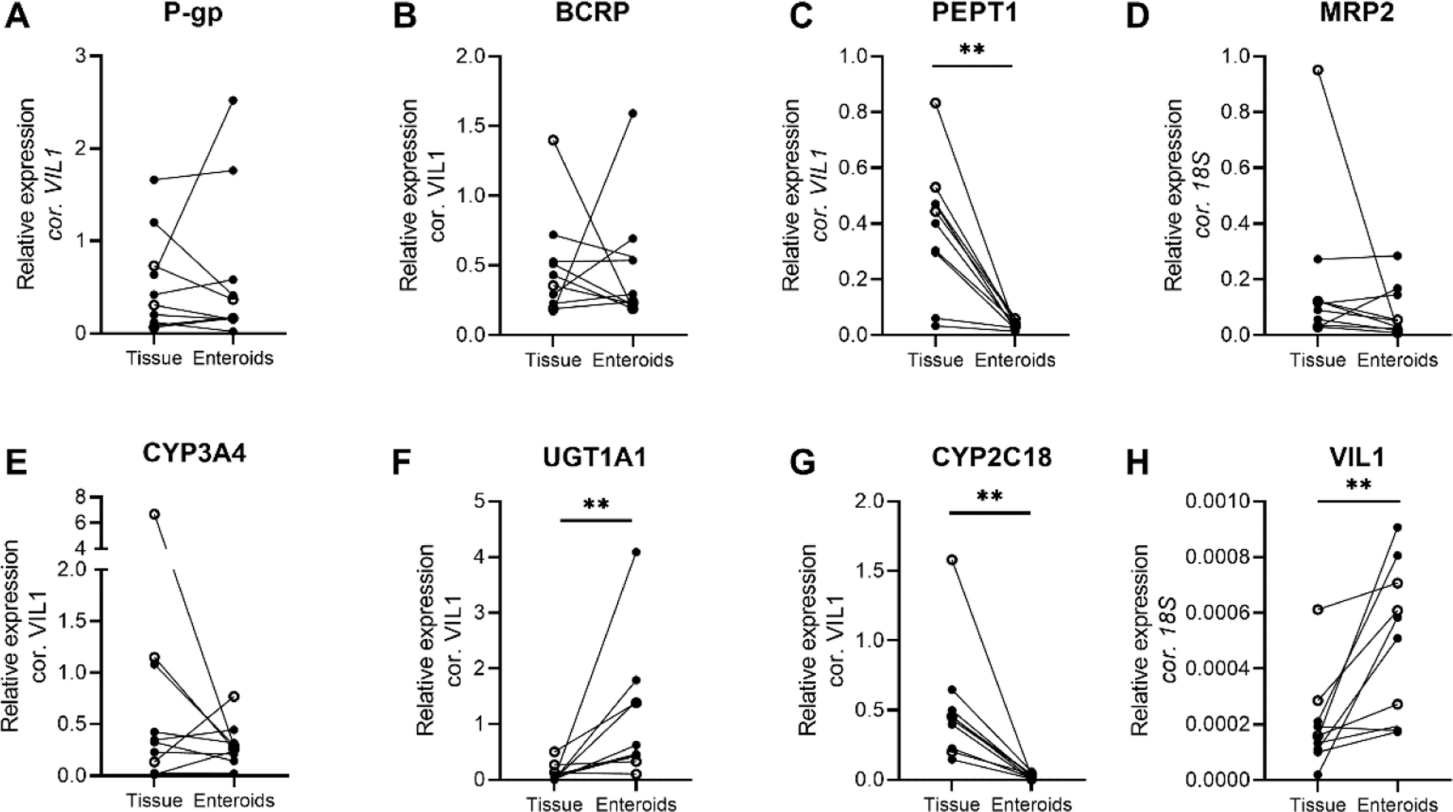
Gene expression in all tissues and enteroid paired donor samples relative to Villin-1 (VIL1), Villin-1 relative to 18S. Similar expression in tissue and enteroids is shown for P-gp, BCRP, MRP2, and CYP3A4. PEPT1, UGT1A1, CYP2C18, and VIL1 show deviating expression between tissue and enteroids. Individual dots represent individual donors for tissue and enteroids. Enteroid values are given in median expression of different passages per donor. In Figure S1, individual tissue and enteroid passage numbers for each donor are plotted in a boxplot representation. Open circles indicate adult donors, and closed circles indicate pediatric donors. (A) P-gp (*ABCB1*), (B) BCRP (*ABCG2*), (C) PEPT1 (*SLC15A1*), (D) MRP2 (*ABCC2*), (E) CYP3A4, (F) UGT1A1, (G) CYP2C18, and (H) Villin-1. **: *p* < 0.01, exact *p*-values and median values per group are presented in Table S3. Age-dependent variation of ADME genes in enteroids and tissue.

**Figure 4:**
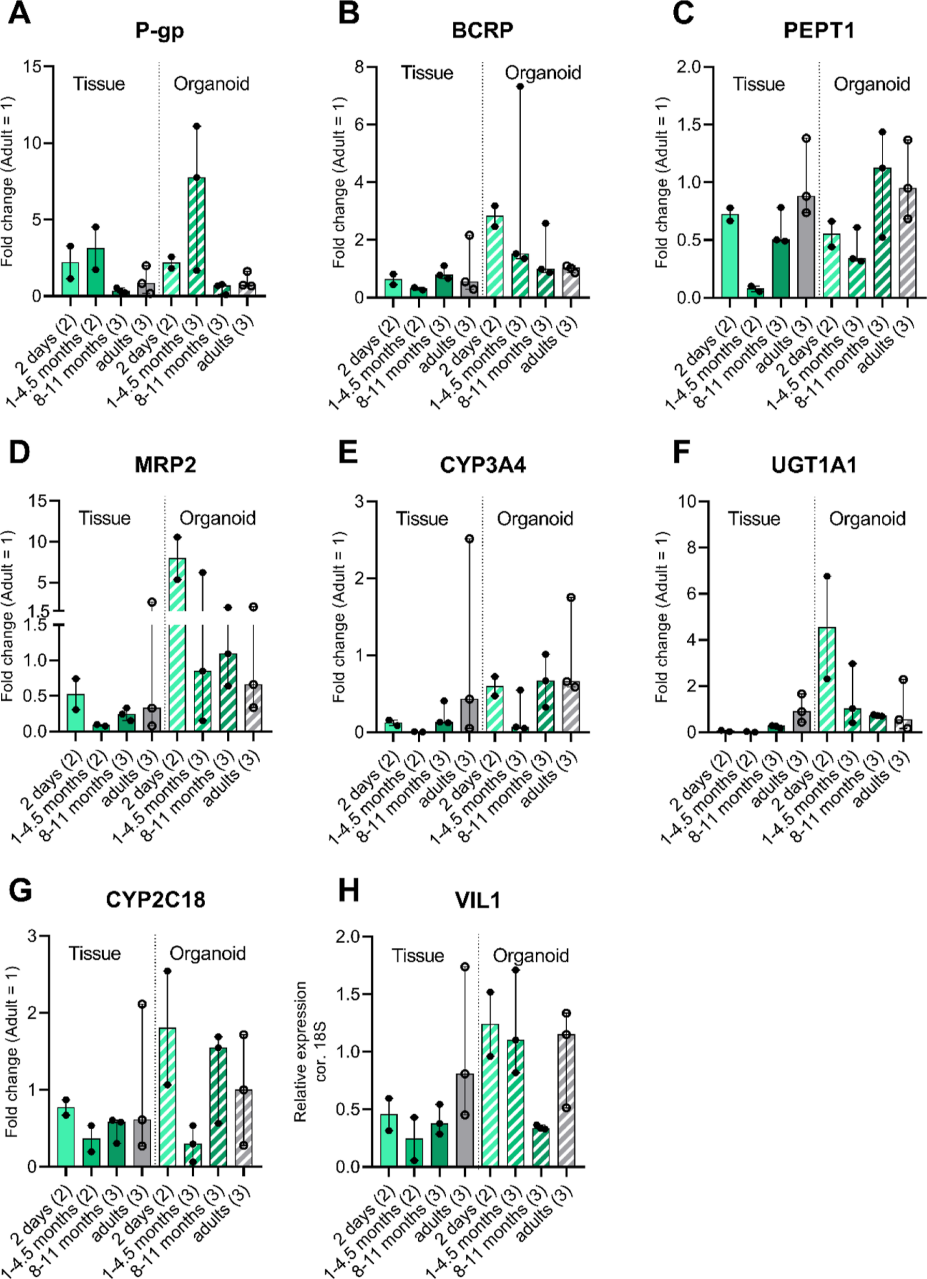
Fold change per gene per age group relative to adults in fresh intestinal tissue and enteroids, showing maturational patterns in tissue and enteroids. Shown in median ± Min. to Max. (A) P-gp (*ABCB1*), (B) BCRP (*ABCG2*), (C) PEPT1 (*SLC15A1*), (D) MRP2 (*ABCC2*), (E) CYP3A4, (F) UGT1A1, (G) CYP2C18, and (H) Villin-1. For enteroids, the dots represent the median of the enteroid passages per donor used in the study. (*n*) is the number of individual donors.

No significance test was performed to test the relationship with age (Figure 4). We visually interpreted Figure 4 for only possible trends. This is due to the low inclusion numbers in this study and variable age range. Pediatric tissue and enteroid gene expressions relative to adults were plotted in the same graph to enable visual comparison of the maturational patterns in the two models.

## Results

### Patient Samples

To determine whether enteroids can be cultured from the intestinal tissue of pediatric donors, surgical leftover intestinal tissue was collected. Residual surgical specimens of the jejunum–ileum transition to terminal ileum from infants (age range: 0.3–45 postnatal weeks, median 10 weeks, and gestational age 24–37 weeks, *n* = 8) were obtained during intestinal surgical procedures. For the adult-derived enteroids, three macroscopically healthy margins from routine hemicolectomies were collected and processed identically (Table 2).

**Table 2:** Patient Characteristics of Included Patients from Which Enteroids Were Isolated^a^

sample ID	post natal age (Weeks)	gestational age (weeks + days)	region	prior diagnosis	surgery type	fresh tissue for RNA analysis	enteroid passage		
							early (P1–P3)	mid (P4–P7)	late (P8–P11)
1	0.29 (2 days)	34 + 4	transition jejunum-ileum	apple peel intestinal atresia	atresia segment resection	yes	P1, P2	P5	NA
2	0.29 (2 days)	34 + 6	ileum	antenatal volvulus	diseased segment resection	yes	P1, P2	P6	NA
3	4.6	24 + 5	ileum	NEC	diseased segment resection	yes	P3	P6	P10
4	6.9	31 + 2	transition jejunum-ileum	NEC	stoma construction	no	P3	NA	NA
5	13.0	31 + 0	terminal ileum	NEC	stoma closure surgery	yes	P2	P6	NA
6	37.4	29 + 1	terminal ileum	meconium plug	stoma closure surgery	yes	NA	P6	P11
7	41.4	37 + 2	terminal ileum	intestinal ischemia	stoma closure surgery	yes	P3	P5	NA
8	45.3	25 + 0	terminal ileum	NEC	stoma closure surgery	yes	P3	P7	NA
9	adult	NA	terminal Ileum	NA	hemicolectomy	yes	NA	NA	P9
10	adult	NA	terminal ileum	NA	hemicolectomy	yes	P1	P7	NA
11	adult	NA	terminal ileum	NA	hemicolectomy	yes	NA	P5	P8

a NA: not applicable; NEC: necrotizing enterocolitis.

### Pediatric and Adult Enteroid Culture

From the collected pediatric and adult intestinal tissues, intestinal crypts were isolated, and enteroids were grown with conventional protocols and buffers. Optically clear, three-dimensional-shaped enteroids were successfully cultured from all tissue samples up to passage 13 over several weeks (Figure 1 and Table 2). The growth and expansion of pediatric and adult enteroids occurred at similar rates (data not shown).

### Stability of Gene Expression over Different Passages

We determined gene expression relative to Villin-1 of all genes of interest at different passages after a continuous enteroid culture to check for gene expression stability during culture and splitting. In Figure 2, the gene expression of all enteroids (pediatric and adult) is sorted on earlier and late passage for the genes of interest. For all genes, relative gene expression was similar between early and late passage numbers (*p*-values in Table S2). Though some individual donors do show a change in gene expression between passage numbers, no relationship with specific donors or passage number was found.

### Relative Gene Expression in Tissue and Enteroids

To answer the research question whether pediatric and adult enteroids reflect the expression pattern of ADME genes compared to the original tissue, the gene expression relative to Villin-1 of the selected panel of genes was determined. P-gp, BCRP, and CYP3A4 were the most abundantly expressed genes in the intestinal tissues, where in enteroids, UGT1A1 was most abundantly expressed (Figure 3A,B,E,F). The absolute expression levels of P-gp, BCPR, MRP2, and CYP3A4 in 3D enteroids were comparable to those in the original intestinal tissue (Figure 3A,B,D,E). The relative expression of PEPT1 and CYP2C18 was uniformly low in enteroids compared to that in the tissue (Figure 3C,G), whereas the expression of UGT1A1 was high in some enteroids compared to the expression in tissue (Figure 3F; *P*-values in Table S3). Relative to 18S, more Villin-1 was present in enteroids compared with intestinal tissue (Figure 3H), indicating the presence of the lamina propria in tissue compared to enteroids. In Figure S1, individual tissue and enteroid passage numbers for each donor are plotted in a boxplot representation.

To explore the relation between ADME gene expression and age, we compared tissues and enteroids derived from donors between 0.3 and 45 postnatal weeks of age (*n* = 8) with those from adults (*n* = 3). As there was no significant effect of passage number on the enteroid gene expression pattern, median relative gene expression values of an enteroid donor over different passages were used for calculation of the fold change in Figure 4. Interindividual variability between donors is present to a similar extent in all defined age groups. Interestingly, for most genes, a similar maturational pattern was visually observed in enteroids compared to that in the original tissues. P-gp, PEPT1, MRP2, CYP3A4, CYP2C18, and Villin-1 appeared to show similar maturational patterns in tissues and enteroids (Figure 4A,C,D,E,G,H). Overall absolute expression levels for these genes were similar for tissues and enteroids, except for CYP2C18 in which overall expression levels were much lower in enteroids than in intestinal tissue (Figure 3). For BCRP and UGT1A1, age-related patterns visible in tissues and enteroids appeared to differ (Figure 4B,F).

## Discussion

We have successfully cultured tissue-derived intestinal enteroids for multiple passages from eight neonatal and infant donors and three adult donors. To determine whether age-specific changes are maintained in enteroids, we quantified and compared the expression of a panel of ADME genes in enteroids and their original tissues. We show comparable gene expression between tissues and enteroids (Figure 3), as well as stable gene expression with increasing passage numbers (Figure 2). Moreover, these enteroids tend to retain the maturational expression pattern of their original tissue for specific DTs and DMEs: P-gp, PEPT1, MRP2, CYP3A4, and CYP2C18 (Figure 4).

To obtain tissue(s) specimens from pediatric patients for application in preclinical ADME research is challenging, especially from children <1 year of age. For intestinal tissue, in contrast to liver and kidney, this is even more challenging, as post-mortem tissue quality is much lower due to fast post-mortem degradation. This, and the small size of surgical resections, limits the ex vivo use of intestinal tissue in activity studies and prompted us to study the feasibility of pediatric enteroids for pediatric ex vivo pharmacokinetic studies. Before, others successfully developed 3D intestinal organoids from pediatric tissue (enteroids). While these enteroids were used to study and treat the underlying disease of interest,^22,23^ our aim was to develop enteroids that specifically represent the age-related variation of ADME genes in gut segments relevant for drug uptake.

Previously, pediatric-derived enteroids have been shown to maintain their DNA methylated pattern corresponding to their age.^13,15^ Recently, pediatric and adult human enteroid monolayer gene expression has been compared using RNA sequencing and morphological studies.^8^ As they hypothesized, enteroids established from infant intestinal tissues reflect characteristics of the immature gut, such as significantly shorter cell height and lower epithelial barrier integrity.^8^ A limitation of their study was that enteroids were not compared to patient-matched fresh tissue expression, and hence, it cannot be concluded if expression levels remain stable from the tissue up to multiple passages in enteroids. Notably, we had a 100% success rate of outgrowing eight infant enteroid lines with commercialized protocols, even in suboptimal (overnight) processing, pointing to the presence of stem cells. Both studies thus clearly point to the feasibility of growing enteroids, even from critically ill infants undergoing surgery for severe intestinal malfunctions. This should prompt further research and establishment of an enteroid derivation infrastructure.

Our study clearly defines advantages and new opportunities of using postnatal enteroids that iPSC organoids do not offer. Postnatal enteroids show more representative expression of PK genes compared to iPSC-derived organoids from the same donor.^24^ Inui et al., 2023, have previously shown that iPSC-derived organoids from adult donors showed PK characteristics that were more comparable to fetal intestinal cells, with a lack of CYP3A4 expression and high CYP2J2 expression compared to biopsy-derived material,^24^ most likely caused by the lack of differentiation of the iPSC-derived intestinal organoids. This is in line with a concept that the environment (drug exposure, food, and microbiota) induces epigenetic changes of epithelia and is not captured in iPSC-derived intestinal cells.^25^ As these influences are unique to each human and drive the large interindividual variability of PK, iPSC-derived organoids are currently not suitable for patient- or cohort-specific ADME and safety aspects and likely other epigenetically regulated mechanisms. Our study adds age-dependent regulation to this concept, although we cannot address the respective contribution of intrinsic regulation versus environmental influence.

In this study, the relative gene expression of the selected panel of ADME genes showed a variation between the different donors. Though some donors do show a change in gene expression between passage numbers or between the tissue and enteroid, no relationship between genes for specific donors or passage number was found. Based on pairwise comparison, the expression remained stable over several passages of enteroid culturing up to at least passage 13. Interestingly, the average expression levels of most genes in enteroids were similar to the expression in corresponding tissues, with the exception of lower PEPT1 and CYP2C18 expressions and higher UGT1A1 expression in enteroids. A possible explanation for the lower PEPT1 and CYP2C18 expressions might be the lower expression of nuclear receptor CAR in enteroids; however, the exact reason remains unclear.^26,27^ For UGT1A1, we have no explanation for the higher expression in the enteroids. The results in this study imply that enteroids may be used to study the relative expressions of ADME genes but not in relation to each other. Next to that, results could indicate that enteroids derived from fresh tissue retain the age-related DT and DME expression patterns of the donor.

The comparison of our data to the ontogeny of the intestinal DT and DME genes of other studies on intestinal tissue is limited by the small sample sizes, different methodologies used (RT-qPCR, proteomics, and Ussing), and the different intestinal regions and age ranges studied. Johnson et al., 2001, showed a maturational pattern for CYP3A4 gene expression in duodenum, with a significant difference for neonates compared to the age groups >5 years.^5^ Lower gene expression in neonates was found for PEPT1 and MRP2 compared to older age groups.^19,20^ Other RT-qPCR studies showed stable P-gp and BCRP expression with age.^20,28^ To the best of our knowledge, intestinal CYP2C18 ontogeny data are lacking.^29^ With the introduction of proteomics, which necessitates only small tissue samples, several groups have studied the age-related intestinal protein expression of relevant DTs and DMEs. Kiss et al., 2021, reported lower ileum protein expression for P-gp, BCRP, PEPT1, and UGT1A1 in 0–2 year-olds (*n* = 29) compared to adults (*n* = 8), while in jejunum, BCRP, CYP3A4, MRP2, and UGT1A1 were significantly lower in 0–2 year-olds (*n* = 8) compared to adults (*n* = 8).^4^ Goelen et al., 2023, did not find expression differences between age groups in duodenum biopsies (youngest group 2–5 years, *n* = 9) for P-gp, BCRP, MRP2, CYP3A4, and UGT1A1, but CYP3A4 increased significantly with age on a continues age scale.^21^ The results by Kiss^4^ and Goelen et al.^21^ are hard to compare with each other due to donor age and intestinal region differences. As most pediatric ontogeny changes are expected during the first year of life, this age group is crucial in age-related effect research.^30^ Finally, activity studies, using the ex vivo Ussing chamber methodology, showed lower mean values for P-gp and BCRP substrate transport across the intestinal barrier compared to adults; however, no correlation with age was found within the pediatric age range (8 weeks −17 years, median: 44 weeks).^6^

There are limitations to this explorative study. We have to consider that all donors were hospitalized and their underlying disease conditions might have had an impact on tissue viability and outcomes found in this study. This is especially true for the four youngest patients, all of whom were ill at the time of the surgery. The intestines of the four older infant donors were considered stable enough to reverse their ileum stoma. Additionally, our donors have been treated with drugs, some of which may have had an influence on gene expression patterns. Higher inclusion numbers are necessary to obtain more robust results and gain more insights into populational variation, as interindividual variability may false-positively be interpreted as ontogeny dependency. Next, gene expression values do not predict protein abundance directly and especially protein functionality. Several publications show the insufficient correlation between RNA and protein expression of DT and DME in tissues.^31−36^ For protein functionality studies in enteroids, the major limitation is the luminal inside representation of the enteroids. To study active drug transport and metabolism of specific substrates inside out, 3D enteroids^37,38^ or monolayer cultures^39−42^ may be better suited, and such data rather than RNA expression may be utilized in in silico drug development. Importantly, the expansion potential of enteroids while retaining ADME expression properties allows us to study whether the maturational characteristics also remain at a functional level. If so, enteroids derived from pediatrics offer a promising platform to investigate not only pharmacokinetics but also toxicity of new drug candidates in this special population.

In summary, we successfully cultured postnatal enteroids from a neonatal pediatric population. In this proof-of-concept study, enteroids show similar expressions of clinically relevant ADME genes compared to tissues. As well as largely similar maturational patterns as the original tissue, there are clinically relevant ADME genes. This study should prompt further research and the establishment of an enteroid derivation infrastructure. Further research is necessary to evaluate if pediatric enteroids may serve as an additional tool in pediatric drug development and in the future may lead to improved pediatric safety predictions during drug development.

## Supporting Information Available

Materials used in cell culture, relative gene expression in all tissues and enteroid samples, and *p*-values of performed statistical tests (PDF)
