# Supplementary material for: Age-Specific ADME Gene Expression in Infant Intestinal Enteroids
Source: Mol Pharm. 2024 Aug 9;21(9):4347–55. doi: 10.1021/acs.molpharmaceut.4c00302 (PMC11372835; doi:10.1021/acs.molpharmaceut.4c00302)
Supplement: Supplementary file 1 — mp4c00302_si_001.pdf [file mp4c00302_si_001.pdf]

Supplementary

Age specific ADME gene expression in infant intestinal enteroids

Eva J. Streekstra[1,2], Tom Scheer-Weijers[1], Michael Bscheider[3], Sabine Fuerst-Recktenwald[3], Adrian B. Roth[3], Sven C.D. van Ijzendoorn[4], Sanne M.B.I. Botden[5], Willem de Boode[6], Martijn Stommel[7], Rick Greupink[1], Frans G. M. Russel[1], Evita van de Steeg[2], Saskia N. de Wildt[1, 8, 9, *]

[1] Department of Pharmacy, Division of Pharmacology and Toxicology, Radboud University Medical Center, Nijmegen, The Netherlands

[2] Department of Metabolic Health Research, Netherlands Organization for Applied Scientific Research (TNO), Leiden, The Netherlands

[3] F. Hoffmann-La Roche Ltd, Basel, Switzerland

[4] Department of Biomedical Sciences, University of Groningen, University Medical Center Groningen, Groningen, The Netherlands

[5] Department of Pediatric Surgery, Radboud University Medical Center, Amalia Children's Hospital, Nijmegen, The Netherlands

[6] Department of Pediatrics, Division of Neonatology, Radboud University Medical Center, Amalia Children's Hospital, Nijmegen, The Netherlands

[7] Department of Surgery, Radboud University Medical Center, Nijmegen, The Netherlands

[8] Department of Intensive Care, Radboud University Medical Center, Nijmegen, the Netherlands

[9] Department of Neonatal and Pediatric Intensive Care, Erasmus MC Sophia Children's Hospital, Rotterdam, the Netherlands

*Corresponding author: Saskia N. de Wildt; Saskia.dewildt@radboudumc.nl. P.O. Box 9101, 6500 HB Nijmegen (Route 137), the Netherlands. Phone number: +31(24)3093477

Supplementary Table 1: composition of solutions used in enteroid isolation and culturing.

Solution	Components	End concentration	Supplier
Storage Buffer	Williams' E		Gibco, Thermo Fisher Scientific, Walthman, MA
	Glucose	2.8 mg/mL	Sigma-Aldrich, Burlington, MA
	Amphotericin B	2.5 µg/mL	Gibco, Thermo Fisher Scientific, Walthman, MA
	Gentamicin	500 µg/mL	Gibco, Thermo Fisher Scientific, Walthman, MA
	GlutaMAX	2mM	Gibco, Thermo Fisher Scientific, Walthman, MA
	HEPES	10mM	Gibco, Thermo Fisher Scientific, Walthman, MA
Wash Buffer	PBS		Gibco, Thermo Fisher Scientific, Walthman, MA
	Pen/Strep	100 U/mL	Gibco, Thermo Fisher Scientific, Walthman, MA
	Gentamicin	500 µg/mL	Gibco, Thermo Fisher Scientific, Walthman, MA
	Amphotericin B	2,5 µg/mL	Gibco, Thermo Fisher Scientific, Walthman, MA
	Primocin	50 µg/mL	Invitrogen, Carlsbad, CA
Crypt releasing solution	Wash buffer		See above
	EDTA	4 mM	Sigma-Aldrich, Burlington, MA
	DTT	2 mM	Roche, Basel, Switserland
Advance DMEM +++	Advanced DMEM		Gibco, Thermo Fisher Scientific, Walthman, MA
	Pen/strep	100 U/mL	Gibco, Thermo Fisher Scientific, Walthman, MA
	GlutaMAX 100x	2 mM	Gibco, Thermo Fisher Scientific, Walthman, MA
	HEPES	10 mM	Gibco, Thermo Fisher Scientific, Walthman, MA
Organoid Growth Medium	IntestiCult™ Organoid Growth Medium (Human)		STEMCELL™ Technologies, Vancouver, BC, Canada
	IntestiCult™ Organoid Supplement		STEMCELL™ Technologies, Vancouver, BC, Canada
	Pen/Strep	100 U/mL	Gibco, Thermo Fisher Scientific, Walthman, MA
	Rock-inhibitor (Y-27632)*	10 µM	STEMCELL™ Technologies, Vancouver, BC, Canada
Organoid Differentiation Medium	IntestiCult™ Organoid Differentiation Medium (Human)		STEMCELL™ Technologies, Vancouver, BC, Canada
	IntestiCult™ Organoid Supplement		STEMCELL™ Technologies, Vancouver, BC, Canada
	Pen/Strep	100 U/mL	Gibco, Thermo Fisher Scientific, Walthman, MA
	DAPT	5 µM	STEMCELL™ Technologies, Vancouver, BC, Canada

*Only added during the first 3 days after enteroid isolation.

Supplementary Table 2: statistical values of Figure 2, Wilcoxon matched-paired signed rank test. IQR: interquartile range.

	Median ± IQR early passage	Median ± IQR late passage	p-value paired donors early vs late passage
**P-gp**	0.47 ± 1.36	0.26 ± 0.46	0.3594
**BCRP**	0.37 ± 0.549	0.27 ± 0.44	0.0547
**PEPT1**	0.028 ± 0.02	0.03 ± 0.03	0.6523
**MRP2**	0.04 ± 0.25	0.05 ± 0.12	0.2500
**CYP3A4**	0.31 ± 0.34	0.21 ± 0.23	0.4258
**UGT1A1**	0.99 ± 1.71	0.69 ± 1.32	0.9102
**CYP2C18**	0.02 ± 0.02	0.02 ± 0.05	0.2500
**VIL-1**	0.0006 ± 0.0008	0.0004 ± 0.0003	0.2500

Supplementary Table 3: statistical values of Figure 3, Wilcoxon matched-paired signed rank test. IQR: interquartile range.

	Median ± IQR tissue	Median ± IQR enteroid	p-value paired donors tissue vs enteroid
**P-gp**	0.36 ± 0.74	0.27 ± 0.72	>0.9999
**BCRP**	0.39 ± 0.36	0.27 ± 0.39	0.7695
**PEPT1**	0.42 ± 0.25	0.03 ± 0.03	0.0020
**MRP2**	0.10 ± 0.12	0.04 ± 0.13	0.1934
**CYP3A4**	0.34 ± 0.99	0.27 ± 0.16	0.2754
**UGT1A1**	0.07 ± 0.16	0.54 ± 1.10	0.0039
**CYP2C18**	0.44 ± 0.32	0.024 ± 0.03	0.0020
**VIL-1**	0.0002 ± 0.00012	0.0005 ± 0.0005	0.0039

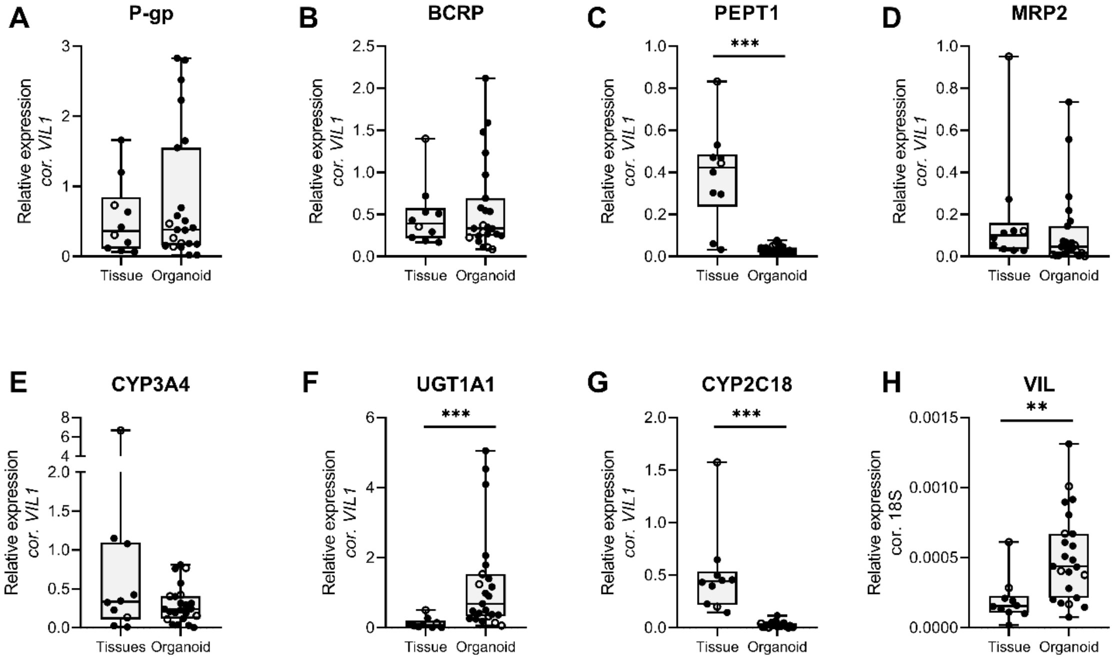

Supplementary Figure 1: relative gene expression in all tissues and enteroid samples corrected for Villin-1 (VIL1), Villin-1 corrected for 18S. Shown in Median ± Min. to Max. Shows similar expression in tissue and enteroids for P-gp, BCRP, MRP2, CYP3A4. PEPT1, UGT1A1, CYP2C18 and VIL1 show deviating expressions between tissue and enteroids. Individual dots represent individual donors for tissue and individual passages of all donors for enteroids. Open circles indicate adult donors, closed circles pediatric donors. A: P-gp (ABCB1), B: BCRP (ABCG2), C: PEPT1 (SLC15A1), D: MRP2 (ABCC2), E: CYP3A4, F: UGT1A1, G: CYP2C18, H: Villin-1. **: p < 0.01, ***: p < 0.001, exact p-values are presentaed in Supplementary table 4.

Supplementary Table 4: statistical p-values of Supplementary Figure 1, Mann-Whitney test

	Early vs late passage
**P-gp**	0.7727
**BCRP**	>0.9999
**PEPT1**	<0.0001
**MRP2**	0.1928
**CYP3A4**	0.3633
**UGT1A1**	0.0001
**CYP2C18**	<0.0001
**VIL-1**	0.0021
